# Evaluating the Role of Urine Cytology in Haematuria One-Stop Clinics: A Scoping Review of Current Evidence and Clinical Utility

**DOI:** 10.7759/cureus.96853

**Published:** 2025-11-14

**Authors:** Oluseyi H Ogunfusika, Aretha Akinluyi, Amgad Elmadani, Tagelsir Saeed, Ayodeji O Adewoye

**Affiliations:** 1 Urology, Kettering General Hospital, Kettering, GBR; 2 Geriatrics, Leicestershire Partnership NHS Trust, Leicester, GBR; 3 Urology, University Hospitals of Northamptonshire, Kettering, GBR; 4 Urology, Norfolk and Norwich University Hospital, Norwich, GBR; 5 Accident and Emergency, Kettering General Hospital, Kettering, GBR

**Keywords:** bladder cancer, cytoscopy, haematuria one-stop clinic, non-visible haematuria, urine cytology, visible haematuria

## Abstract

Urine cytology has traditionally been part of haematuria assessment pathways for detecting high-grade urothelial carcinoma, particularly carcinoma in situ (CIS), but its value within modern Haematuria One-Stop Clinics (HOSCs) is increasingly debated in light of new diagnostic approaches, economic pressures, and updated international guidelines. This scoping review takes into account current evidence and recommendations from the American Urological Association (AUA), European Association of Urology (EAU), British Association of Urological Surgeons (BAUS), and National Institute for Health and Care Excellence (NICE), drawing on four extensive peer-reviewed studies to assess the diagnostic accuracy, clinical relevance, and cost-effectiveness of urine cytology in one-stop haematuria models. Across all major guidelines, there is consensus that urine cytology should not be routinely used in the initial evaluation of haematuria, with selective use reserved for patients at risk of CIS or with equivocal cystoscopy findings. While cystoscopy and cross-sectional imaging remain the principal diagnostic tools, variation in practice persists, particularly in the UK, due to established custom, resource limitations, and medico-legal considerations. Urine cytology retains a limited, targeted role in detecting high-grade disease, but its routine use in HOSCs lacks robust supporting evidence; standardising practice in line with international guidance will promote risk-stratified, cost-effective, and consistent haematuria management.

## Introduction and background

The development of Haematuria One-Stop Clinics (HOSCs) closely followed the United Kingdom’s goal to improve cancer services. This goal was highlighted by the launch of the Two-Week Wait (2WW) referral pathway in 2000, following the government's 1997 White Paper, The New NHS: Modern, Dependable [1]. The 2WW pathway required that patients suspected of having cancer be seen by a specialist within two weeks of their first consultation. This effort was part of a broader national strategy to lower cancer deaths by promoting earlier diagnosis and prompt treatment. In the context of haematuria, the 2WW pathway allowed for quick referrals to urology services and emphasised the need to rule out cancer, especially bladder cancer.

Haematuria can be classified as visible (VH) or non-visible haematuria (NVH). Non-visible or microscopic haematuria is defined as the presence of three or more red blood cells per high-power field [2]. However, NVH in the UK is confirmed by a positive urine dipstick test. The National Institute for Health and Care Excellence (NICE) has recommended, in its guidelines, a referral via the 2WW for patients aged 45 and above with visible haematuria in the absence of infection, and for those aged 60 and above with NVH and either a raised white cell count or dysuria [3]. Haematuria is a common presenting symptom for urinary tract malignancy, most frequently bladder cancer. However, it can also stem from benign conditions such as urolithiasis, infections, benign prostatic hyperplasia, and nephrological causes [4].

The development of HOSCs evolved from a synergy between the mandate described in the 1997 white paper and the NICE 2WW referral pathways. Major landmark studies of HOSCs in the UK were those of Edwards et al. [5] and Khadra et al. [6], both of which drew on data from large patient groups attending the HOSC. For example, in their study of over 4,000 cases, Edwards and his team found bladder cancer in 18.9% of patients with visible haematuria and in 4.8% of those with non-visible haematuria.

A typical HOSC assessment includes a same-day evaluation using cystoscopy and imaging, which may be ultrasound or computed tomography (CT), depending on the patient's risk profile. Risk assessment considers factors like smoking history, work-related exposure, such as contact with aromatic hydrocarbons, heavy metals, and family history of urothelial malignancy [7]. Urine cytology is often debated in the diagnostic process and is generally considered a weak adjunct to cystoscopy [8]. It is still carried out as part of the HOSCs across the UK. This study focuses on voided urine cytology, which is known to yield more reliable results than instrumented samples [9].

Despite minor variations among international urological guidelines, there is now consensus that urine cytology should not form part of the initial evaluation of haematuria; this position is reflected in the current NICE guidance [10]. At the same time, the British Association of Urological Surgeons (BAUS) aligns with NICE on the role of urine cytology in haematuria workup. In the past, the American Urological Association (AUA) recommended cytological testing for patients with persistent NVH after negative evaluations or for those with risk factors for carcinoma in situ (CIS). However, the updated 2025 AUA guideline now advises against using urine cytology in the initial assessment of both low- and high-risk patients [11]. This study aims to review the existing literature on extensive studies of the HOSC, to determine whether there is evidence supporting the continuous use of urine cytology in the HOSC, or whether urine cytology is now a relic. In addition, various national urology guidelines will be explored and outcomes compared. 

## Review

Study design

This scoping review aims to evaluate the role of urine cytology in HOSCs, focusing on its diagnostic utility, limitations, and clinical relevance. A systematic literature review was conducted to identify studies exploring the use of urine cytology in the evaluation of haematuria, with particular emphasis on the one-stop clinic model.

Eligibility criteria

We included studies that address the diagnostic utility of urine cytology in patients with haematuria, with or without a focus on one-stop clinics. Report clinical outcomes or findings related to the use of urine cytology in the diagnostic workup of haematuria, including its sensitivity, specificity, and role in identifying malignancies (e.g., bladder cancer). These include primary research studies such as randomised controlled trials (RCTs), cohort studies, case-control studies, cross-sectional studies, and other relevant observational studies. They were published in English between 2000 and 2025.

Exclusion criteria

Studies that did not focus on haematuria or urinary tract malignancies, especially in the setting of the HOSC, case reports, editorials, and opinions were excluded.

Studies involving haematuria investigations that do not use the three modalities of flexible cystoscopy, urine cytology, and imaging, such as CTU (computerised tomography urography) or ultrasonography, and articles that did not provide relevant data on urine cytology or the one-stop clinic model were excluded.

A comprehensive literature search was performed using the following databases: PubMed (National Library of Medicine, NLM), EMBASE (via the Ovid platform), Medline and the Knowledge & Library Hub (via the EBSCO platform), as well as Google Scholar. The search was conducted using keywords and Medical Subject Headings (MeSH) terms relevant to the topic, such as: “Urine Cytology”, “One-Stop Clinics”, “Bladder Cancer” “, Diagnostic Utility” “, Cystoscopy” “, Haematuria” “, Urinary Tract Malignancy”

There were minimal studies exploring HOSCs in patients undergoing strictly flexible cystoscopy, imaging (ultrasonography/CT), and urine cytology. Four extensive studies were isolated. 

Characteristics of studies 

Viswanath et al. (2008) [12] conducted a prospective study evaluating 1,000 consecutive patients attending a one-stop haematuria clinic at the Norfolk and Norwich University Hospital. Both microscopic and macroscopic haematuria cases were included. All patients underwent urine cytology, upper tract imaging, and flexible cystoscopy. Munro et al. (2010) [13] conducted a three-year prospective evaluation of a visible haematuria clinic; this study assessed the role of cytology alongside standard investigations. Blick et al. (2011) [14] was a multicentre diagnostic accuracy study that evaluated 778 patients investigated for haematuria using CT urography, flexible cystoscopy, and voided urine cytology, and Tan et al. (DETECT I) 2019 [15] was an extensive multicentre prospective observational study involving 3,556 haematuria patients across 40 UK hospitals. 

Results 

Urine cytology has long been used to evaluate haematuria and detect urothelial carcinoma, but its role in the initial diagnostic pathway remains controversial. This paper synthesises findings from Viswanath et al. (2008) [12], Munro et al. (2010) [13], Blick et al. (2011) [14], and Tan et al. (2019) [15] to assess the diagnostic, clinical, and economic implications of routine cytology in haematuria clinics.

Data from the four extensive studies demonstrate highly consistent patterns: specificity remains high (90-98%), but sensitivity ranges from moderate to low (38-66%), depending on the definition of "atypical" interpretation.

There is unanimous agreement among studies that cytology rarely detects urothelial malignancies occult to cystoscopy or modern imaging techniques such as CT scans. Curiously, in the most extensive multicentre data set (DETECT I), 52.3% of bladder cancers diagnosed had a false-negative result, including some of the higher-risk tumours. Munro et al. showed that cytology yielded 42 false positives (11% of those assessed in the cohort), and no occult tumours were diagnosed after three years of follow-up [13].

**Figure 1:**
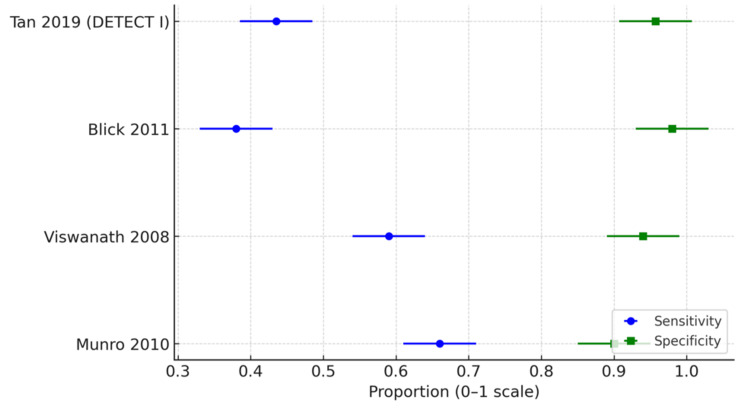
Proportion of urine cytology showing sensitivity vs specificity across the four studies Forest plot of urine cytology sensitivity and specificity in haematuria evaluation studies

**Figure 2:**
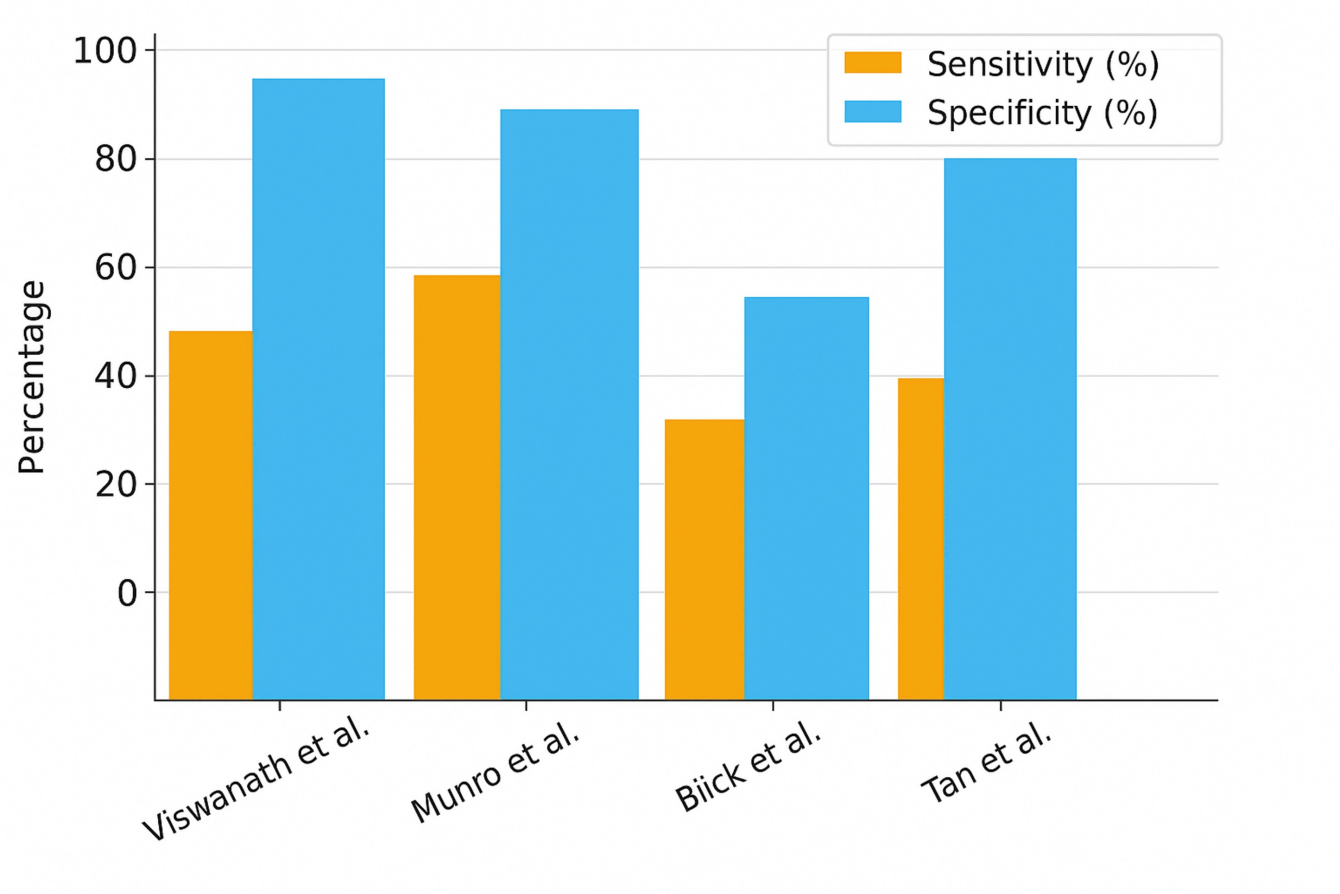
Urine cytology distribution across the studies

Viswanath et al. found it challenging to justify urine cytology as a tool in the haematuria workup [12]. In their series of over 1000 cases of haematuria, no TCC (transitional cell carcinoma) diagnosis was made based on urine cytology alone. See Figures 1, 2 for sensitivity and specificity of urine cytology across the studies.

These observations as shown in Table 1 collectively call into question the rationale for the universal use of cytology in patients attending one-stop haematuria clinics. For both prospective and retrospective groups, detection of abnormal cytology comparatively seldom enhanced diagnostic yield but oftentimes precipitated further assessments through cystoscopy or imaging with minimal gain. False-positive records impose unnecessary procedural, psychological, and economic burdens upon patients and health care systems. By contrast, the consistently good negative predictive value (84-95%) indicates a low disease prevalence rather than actual diagnostic reassurance; a cytologic finding of negativity cannot exclude malignancy.

**Table 1:** Comparative and summary table of urine cytology in haematuria clinics across the studies CTU: Computerised tomography urography; TCC: transitional cell carcinoma

Study (Year)	Setting	Sample size analysed	Design	Primary test(s)	Cytology sensitivity (%)	Cytology specificity (%)	PPV (%)	NPV (%)	Key outcomes	Follow‑up
Viswanath et al. (2008)	One‑stop haematuria clinic (micro + macro)	986	Prospective consecutive cohort	Urine cytology + imaging + flexible cystoscopy	59.0	94.0			No malignancy diagnosed by cytology alone; abnormal cytology → further tests; none later returned with TCC; total cost ~£50,535	Clinic records; no subsequent TCC among false positives reported
Munro et al. (2010)	Visible haematuria clinic	503	Retrospective cohort with 3‑year follow‑up	Urine cytology + ultrasound + flexible cystoscopy	66.0	90.0	50.0	94.0	All bladder tumours found by cystoscopy; 3 upper‑tract TCC by ultrasound; cytology found no extra tumours; no occult cancers at 3 years	3 years (median ~41.5 months)
Blick et al. (2011)	Hospital haematuria rapid diagnosis clinic	747	Prospective diagnostic accuracy cohort	CT urogram + flexible cystoscopy + voided cytology	38.0	98.0	82.0	84.0	Optimal strategy: CT urogram + flexible cystoscopy; cytology sensitivity too low for routine use	21–66 months reference standard verification
Tan et al. (DETECT I, 2019)	Multicentre UK (40 hospitals) haematuria pathways	567	Prospective observational study	Urine cytology + RBUS/CTU + cystoscopy	43.5	95.7	47.6	94.9	Cytology missed 21 bladder + 5 UTUC (incl. ≥pT2); adding CTU → sensitivity ~90%; routine cytology not recommended	≥1 year for false positives/negatives subgroup

Methodological Variations

Methodological variations, such as single versus multicentre design and differing cytology thresholds, account for some numerical differences between studies; however, their conclusions converge on the same clinical point. The diagnostic value of cytology is redundant to a considerable extent when flexible cystoscopy and computerised tomography urography (CTU) are used routinely. Selective application still has a role in certain situations: carcinoma in situ suspicion, high-grade tumour follow-up, or when the findings are uncertain at cystoscopy. Combined CTU and cytology approaches may add a little to sensitivity but must be weighed against the added cost and false-positivity. Tan et al. (DETECT 1) recognise the dilemma of false-positive urine cytology with imaging and flexible cystoscopy negative. False positives often lead to repeat imaging and invasive testing. The DETECT I study found that over half of patients with false-positive cytology underwent unnecessary procedures, none of which revealed malignancy; hence it does not recommend follow-up.

Another emerging theme is cost-effectiveness and resource implications: routine urine cytology incurs high laboratory and downstream costs with limited diagnostic yield. Viswanath et al. estimated a total of £50,535 for cytology-related procedures, with no malignancy identified solely by this test, accounting for up to 20% of the cost of HOSC per patient.

Integration with Imaging Modality

**Figure 3:**
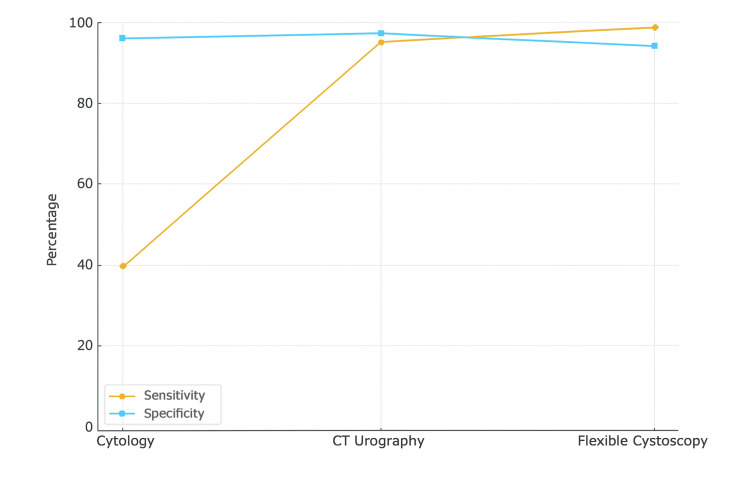
A graph comparing the sensitivity and specificity of CTU, urine cytology and flexible cystoscopy based on the report across the studies CTU:  Computerised tomography urography

Across all reviewed studies, urine cytology demonstrated consistently high specificity but limited sensitivity. No study identified additional urothelial malignancies beyond those detected by cystoscopy or cross-sectional imaging, highlighting its restricted diagnostic utility as an independent investigative modality. The studies also unanimously agree that combining CT urography with flexible cystoscopy achieved a near-perfect diagnostic accuracy, whereas cytology alone performed poorly. Cytology may have only supplementary value when used adjunctively, for example, in a patient with CIS (carcinoma in situ). See Figure 3 for a description.

Current evidence demonstrates that traditional urine cytology provides minimal ancillary diagnostic assistance in the evaluation of haematuria. It is advisable that recent and forthcoming guidelines advocate a selective, risk-dependent strategy rather than a mass strategy, ensuring cytology has a paramount role in high-yield scenarios where its specificity yields significant clinical benefits.

What Do the Guidelines Say?

The European Association of Urology (EAU) distinctly categorises cytology as a supplementary tool to cystoscopy in scenarios where high-grade malignancy is suspected. It advocates standardised reporting in accordance with The Paris System, a reporting standard for both voided and instrumented samples [16]. EAU also emphasises that cystoscopy remains irreplaceable by cytology or alternative non-invasive methodologies. Within the EAU diagnostic framework, cytology is endorsed to facilitate the identification of high-grade neoplasms, while the preliminary assessment of haematuria prioritises cystoscopy and upper-tract imaging [17].

In the United Kingdom, the British Association of Urological Surgeons (BAUS) has retracted its previous independent recommendations on haematuria. It now defers to the National Institute for Health and Care Excellence (NICE). The NICE referral guideline [3] delineates the criteria for urgent investigation of suspected malignancy, whereas its bladder cancer guideline downplays the role of cytology, advising against the routine application of urinary cytology in low-risk scenarios and instead emphasising endoscopic evaluation and imaging; this perspective is consistent with empirical evidence indicating a limited diagnostic yield associated with routine cytology in haematuria clinics. NICE emphasises the importance of a systematic approach, including an initial assessment, appropriate imaging, and prompt referral to specialist services. The NICE guidelines support the use of HOSCs as a model of care consistent with these principles and promote their implementation to provide evidence-based, effective and patient-centred care. Together with these, the European Association of Urology (EAU) offers detailed recommendations for the treatment of haematuria.

The American Urological Association (AUA) microhaematuria guideline (2020, revised 2025) similarly counsels against the routine implementation of cytology for initial assessment, advocating for its reserved application in specifically identified higher-risk situations or in cases of persistent irritative symptoms, while also introducing conditional utilisation of urinary biomarkers for intermediate-risk patients who have been adequately counselled; summaries additionally indicate that cytology is frequently obtained during the evaluation of gross haematuria [18].

## Conclusions

In conclusion, a review of predominant guidelines indicates that the utilisation of urine cytology in the initial evaluation of haematuria is limited; the EAU advocates for its use as a supplementary tool in cases where high-grade malignancy is anticipated; the NICE and the BAUS do not endorse its routine application, especially in a setting that applies to the HOSC. Lastly, the AUA clearly does not recommend it in the setting of HOSC; however, it recommends a selective, risk-adjusted approach to UC, suggesting consideration of biomarkers in specified scenarios. The practical ramifications for secondary urology care clinics in the United Kingdom are that cystoscopy, supported by appropriate imaging, will continue to be the fundamental approach to the workup of patients with visible and non-visible haematuria.
